# Measurements of Radiofrequency Radiation with a Body-Borne Exposimeter in Swedish Schools with Wi-Fi

**DOI:** 10.3389/fpubh.2017.00279

**Published:** 2017-11-20

**Authors:** Lena K. Hedendahl, Michael Carlberg, Tarmo Koppel, Lennart Hardell

**Affiliations:** ^1^Independent Environment and Health Research Luleå, Sweden; ^2^Department of Oncology, Faculty of Medicine and Health, University Hospital, Örebro, Sweden; ^3^Department of Work Environment and Safety, Tallinn University of Technology, Tallinn, Estonia

**Keywords:** radiofrequency radiation, wireless fidelity, Wi-Fi, exposimetric measurements, schools, children, health

## Abstract

**Introduction:**

Wireless access to the Internet is now commonly used in schools. Many schools give each student their own laptop and utilize the laptops and wireless fidelity (Wi-Fi) connection for educational purposes. Most children also bring their own mobile phones to school. Since children are obliged by law to attend school, a safe environment is important. Lately, it has been discussed if radiofrequency (RF) radiation can have long-term adverse effects on children’s health.

**Method:**

This study conducted exposimetric measurements in schools to assess RF emissions in the classroom by measuring the teachers’ RF exposure in order to approximate the children’s exposure. Teachers in grades 7–12 carried a body-borne exposimeter, EME-Spy 200, in school during 1–4 days of work. The exposimeter can measure 20 different frequency bands from 87 to 5,850 MHz.

**Results:**

Eighteen teachers from seven schools participated. The mean exposure to RF radiation ranged from 1.1 to 66.1 µW/m^2^. The highest mean level, 396.6 µW/m^2^, occurred during 5 min of a lesson when the teacher let the students stream and watch YouTube videos. Maximum peaks went up to 82,857 µW/m^2^ from mobile phone uplink.

**Discussion:**

Our measurements are in line with recent exposure studies in schools in other countries. The exposure levels varied between the different Wi-Fi systems, and if the students were allowed to use their own smartphones on the school’s Wi-Fi network or if they were connected to GSM/3G/4G base stations outside the school. An access point over the teacher’s head gave higher exposure compared with a school with a wired Internet connection for the teacher in the classroom. All values were far below International Commission on Non-Ionizing Radiation Protection’s reference values, but most mean levels measured were above the precautionary target level of 3–6 µW/m^2^ as proposed by the Bioinitiative Report. The length of time wireless devices are used is an essential determinant in overall exposure. Measures to minimize children’s exposure to RF radiation in school would include preferring wired connections, allowing laptops, tablets and mobile phones only in flight mode and deactivating Wi-Fi access points, when not used for learning purposes.

## Introduction

In recent years, the education in many schools has changed considerably, from classes where the teacher holds lectures in front of a class, where every student has his/her own school books to read and write in, to classes where all students use their own laptop and no printed text books. The teacher may act as a mentor in the individually based education where the students work at their own pace and capacity directly through different programs on their personal laptops. Access to the Internet is, in most schools, available through wireless connection, wireless fidelity (Wi-Fi). There is usually a network of access points for Wi-Fi in classrooms and corridors to make it possible to teach and keep in contact everywhere in the school building. Most students and teachers also bring their own mobile phones, usually a smartphone, with them to class and use them for social communication and entertainment throughout the day. Besides Wi-Fi, other mobile networking protocols are also actively used—subscription to 3G and 4G wireless connections find widespread use among school children.

Information and communication technology (ICT) is an important field in teaching in our schools, both for every student to learn how to get information in different subjects and as a tool to learn and practice knowledge. Most students and teachers appreciate the development of more technology in school education and are very positive to all advantages it brings.

However, the benefits of ICT in education seem to be largely unproven and there are also concerns that there may be side effects (1). Results from the OECD’s PISA performance surveys from 2012 in reading and mathematics show decreasing results in countries that have invested the most in introducing computers in schools. Sweden is the country that deteriorated the most in the PISA performance surveys in mathematics and reading between 2003 and 2012. At the same time, Sweden was one of the countries that had invested the most in introducing computers in school (2). Results from the PISA performance surveys 2015 from Sweden in reading and mathematics show better results and are now just above the OECD average. However, Sweden is still well below its scores in 2003 (3).

Multitasking, to do several things at the same time, means that your attention has to shift focus between different activities. The brain can only concentrate on one matter at the time. Swift shifting of focus between different tasks may lead to impaired ability to store memories and knowledge (4–7). Even to have nearby classmates surfing on the Internet can impair learning (8). The time used to check their smartphone for new messages can give a positive feeling of being updated but takes time and concentration from school work (9).

Apart from during school time, most students use their smartphones, laptops, and tablets during evenings for several hours. In the yearly inquiry 2015/2016 in the schools in the county of Norrbotten in Sweden, 80% of the 16-year-old students spent at least 3 h in front of a screen in their free time. Five hours or more were spent by 35% (10). If the students also use a laptop during most classes in school, it can add up to many hours of screen time every day. A study in the United States concluded that college students spent nearly 9 h a day on their cell phones (11).

Screen time can affect us in many different ways. Long hours of sitting in front of a screen can lead to aches in the neck, back, and shoulders. In the yearly inquiry in Norrbotten, almost one-fourth of the 16-year-old girls often had aches in their neck, back, or shoulder (10).

Screen time has been shown to have a higher correlation to overweight and obesity than lack of physical activity (12), since prolonged sedentary activities can be a metabolic risk factor (13) and less physical activity may reduce cardiorespiratory fitness (14). There are also concerns about the emotional development for especially young children with a high amount of screen time. Time is taken from play, physical activities, and being with friends, parents, and siblings, which are important parts when growing up. The development of mirror neurons in the brain, which give us the possibility to imitate the behavior of other people, can be affected (15). Empathetic concern among American college students has dropped sharply since 1979 and especially since the year of 2000 according to a meta-analysis on studies performed between 1979 and 2009 (16).

High ICT usage among young adults in Gothenburg led to an increased risk for depression, sleep problems, and stress (17). Among adolescents in Japan using a mobile phone after lights out at night gave sleep disturbances, increased tiredness during day time, and several had suicide thoughts and worse mental health (18, 19). In a review, bedtime access and use of portable screen-based media devices showed a statistically significant association with inadequate sleep quality, poor sleep quality, and excessive daytime sleepiness (20). Increased duration of mobile phone use has been linked to higher risks for depressed mood and mobile phone addiction (11, 21, 22).

Adolescents at risk for mental health problems showed an association between both the amount of time spent using digital technologies and the numbers of text messages sent and increased same-day symptoms from attention deficit hyperactivity (ADHD) and conduct disorders (23). One study in Switzerland raised the question “whether problematic mobile phone use is the consequence of unfavorable conditions or whether and to what extent problematic mobile phone use reinforces behavioral problems as well as decreased mood and psychological well-being” (24).

In the WHO study, Health Behavior in School-aged Children (HBSC) conducted during 2013/2014 results from Sweden showed that girls’ mental health has deteriorated compared with the same study made during 2009/2010. The study has since 1985/1986 taken place around the world every fourth year among 11-, 13-, and 15-year-old girls and boys in over 40 different countries. In Sweden, sleep problems in particular have increased among 13- and 15-year-old girls. Of the girls, 39% have sleep problems more than once a week. Also, boys at the same age have sleeping problems, though to a lesser extent (around 27%). To feel depressed, nervous, or irritated has increased among Swedish, 13- and 15-year-old girls and were about twice as common compared with boys at the same age. The conclusion of the HBSC report brings up that “the fast technical development has changed children’s and adolescents’ everyday life in many ways, which may have influenced their mental well-being” (25). In the latest yearly inquiry in Norrbotten, Sweden, there is a statistically significant correlation between school districts with high amount of screen time and how many students in the community that feel depressed (10).

Considering the long hours of screen time and deteriorating health with increasing problems with sleep, depression, and aches among students, there are concerns regarding whether radiofrequency (RF) radiation from wireless networks, laptops, and mobile phones is harmless below reference values of RF radiation and if there can be potential adverse health effects from this exposure in the long term.

The reference values for RF radiation 10 MHz to 300 GHz were recommended in 1998 by the International Commission on Non-Ionizing Radiation Protection (ICNIRP) to 2–10 W/m^2^ depending on frequency (26). This reference value protects against injuries caused by a heating effect over 1°C after an exposure of 30 min, and with a safety factor of 50. Injuries caused by other biological mechanisms than heating or from chronic effects of RF radiation are not taken into account in the ICNIRP’s reference values. Sweden and many other countries apply the reference value of 10 W/m^2^ (10,000,000 µW/m^2^) for frequencies 2–300 GHz. Some countries, like Russia, Poland, Italy, India, and China have chosen lower reference values down to 0.1 W/m^2^ (100,000 µW/m^2^) (27). In 2012, the Bioinitiative Working Group proposed a precautionary target level of 3–6 µW/m^2^, with a safety factor of 10, since research studies on biological effects have shown biological effects of RF radiation down to 30–60 µW/m^2^ (28).

The Council of Europe recommended in a resolution from 2011 that schools and other buildings where children spend their time should give preference to wired Internet connections and implement information and awareness-raising campaigns on the risks of potentially harmful long-term biological effects on human health from wireless technology, especially targeting children, teenagers, and people of reproductive age (29). In France, mobile phones are not allowed up to sixth grade in school. Israel and Cyprus have recommendations to not have wireless networks in preschool and to turn off Wi-Fi when not used in primary schools. Many schools in Sweden have started to ban mobile phones during school time since they disturb the students’ concentration on school work.

Exposure to RF radiation was classified as a possible human carcinogen, Group 2B, by the International Agency for Research on Cancer (IARC) at WHO in 2011. The decision was mainly based on case–control human studies on use of wireless phones by the Hardell group from Sweden and the IARC Interphone study, which showed increased risk for brain tumors, i.e., glioma and acoustic neuroma (30–33). Further research has confirmed the increased risk for brain tumors and mobile phone use (34–37). A report released in 2016 from the National Toxicology Program under the National Institutes of Health in the United States strengthened the association between RF radiation and cancer. An increased incidence of glioma in the brain and malignant schwannoma in the heart was found in life-long RF radiated rats (38), thereby supporting human epidemiological studies on brain tumor risk. Several laboratory studies have shown mechanistic effects in carcinogenesis such as oxidative stress, down regulation of mRNA and DNA damage with single strand breaks (39–43).

The risk for cancer may be accentuated for children partly because of their likely longer life-time use of wireless devices, but also since their smaller size and thinner skull bone give higher RF radiation to the brain (44). Children are also growing and have more immature cells which can be more sensible to RF radiation (41, 45).

Beside the cancer risk, studies with laboratory animals have shown that RF radiation may open the blood–brain barrier and may thereby increase the rate toxic molecules enter the brain tissue, hurt neurons in the hippocampus (the brain center for memory), impair spatial memory in exposed rats (46–49), and down or up regulate essential proteins in the brain engaged in the brain’s metabolism, stress response, and neuro-protection (50). Long-term, low intensity of RF radiation exposure has also shown reduced levels of neurotransmitters and key-regulating enzymes in the rat brain (51). Increase in frequency seems to have more deleterious effect on several of the parameters (42).

Long-time exposure from a new GSM base station in Rimbach, Germany revealed adverse effects on neurotransmittors like Epinephrine, Norepinephrine, Dopamine, and Phenyletylamine which after 18 months had not normalized, especially not in children and chronically ill adults (52). Chronic dysregulation of psychobiological stress markers may contribute to health problems and chronic illnesses. More behavioral problems have been seen in children with higher exposure to RF radiation compared with children with lower exposure (53–56).

Several studies on humans indicate an influence on the electrical activity in the brain seen in EEGs after exposure to RF radiation during both sleep and in active memory tests (57, 58). Other studies on people exposed to RF radiation from mobile phones have shown disturbed glucose metabolism in the brain (59), effects on endocrine system (60), and in young adults with high-cumulative amounts of hours of mobile phone use, decreased β-trace protein, which is a key enzyme in the synthesis of a sleep-promoting neurohormone (61). DNA damage has also been found in hair root cells after 30 min of mobile phone talk (62) and buccal mucosal cells in high-mobile phone users with more than 10 h of mobile phone use per week for over 5 years (63).

Wireless fidelity exposure on testes and sperms has led to decreased sperm mobility, more head defects, and DNA damage (39, 64–66). Wi-Fi signals in studies on laboratory animals have had effects on heart rhythm and blood pressure after 1 h of exposure (67). Low-intensity long-term exposure to Wi-Fi up to 12 months in animals induced oxidative stress in the lens (68), increase in pro-inflammatory cytokines, oxidative stress, and DNA damage with single strand breaks in the hippocampus in the brain (42), down regulation of microRNA expression in brain tissue (40), and cognitive impairment and DNA damage (69). In humans, Wi-Fi exposure during a language test showed gender-related effects on EEG in a large area of the brain (70), but no effect on EEG or on a psychomotor vigilance test during a 60-min Wi-Fi exposure (71).

It is important to collect information about the RF radiation exposure in schools to consider the best technological devices used in education regarding convenience and economy, but also to consider the potential long-term health effects as mentioned above.

The aim of this study was to assess RF emissions in the classroom by measuring the teachers’ RF exposure and thereby to approximate the children’s exposure but also to identify the main sources of exposure. The investigated schools were equipped with a wireless infrastructure. We wanted to evaluate the measured RF radiation levels in relation to ICNIRPs’ and Sweden’s reference values, but also in relation to countries with stricter safety limits and to the Bioinitiative Working Group’s suggested level. Furthermore, we wanted to compare our measured results with exposimetric studies in schools in other countries. Finally, based on measurements results, we discuss strategies for RF radiation risk management in schools.

## Materials and Methods

This is a descriptive study with measurements of exposure to RF radiation in schools with Wi-Fi and one laptop for each student. School principals were contacted by phone and if interested, information was sent by mail. The principal, or in some cases interested teachers, recruited the teachers willing to participate. All teachers were informed both by written information and personally at the start of measurements by one of the researchers. All teachers signed a statement of agreement to participate before starting the measurements in accordance with the declaration of Helsinki. The Ethical Committee approved the study (Uppsala University DNR 2015/485).

In total, 18 teachers were recruited from seven schools, both rural and urban. The schools were located in the city of Örebro in the middle of Sweden and in the county of Norrbotten in the northern part. The included schools had from 28 to 1,300 students (Table 1). The teachers carried the exposimeter (weight 440 g) in a small and comfortable shoulder bag. The exposimeter was carried during the whole working day. If the teacher also carried a mobile phone on the body, the teacher was advised to place it on the opposite side from the exposimeter. Signals from the mobile phones did not affect the results as the exposimeter is capable of discriminating between upload and download bands. The teachers were carrying the exposimeter from 1 to 4 days.

**Table 1:** Participating teachers and schools.

Teacher	Grades	Number of students	Urban, rural
MH1	7–9	85	Urban
MH2	7–9	85	Urban
MH3	7–9	85	Urban
OH	7–9	111	Rural
SH	7–9	28	Rural
LG1	10–12	170	Rural
LG2	10–12	170	Rural
LG3	10–12	170	Rural
OG1	10–12	190	Rural
OG2	10–12	190	Rural
SG1	10–12	1,300	Urban
SG2	10–12	1,300	Urban
SG3	10–12	1,300	Urban
SG4	10–12	1,300	Urban
TG1	10–12	1,200	Urban
TG2	10–12	1,200	Urban
TG3	10–12	1,200	Urban
TG4	10–12	1,200	Urban

The teachers handed over their work schedule and also filled in a questionnaire about (1) the school building, (2) classroom for each lesson, teachers’ office or lunch room, (3) if there was any access point for Wi-Fi in the room, (4) the number of students in each class, (5) if they used their laptops, (6) if they were connected to the Internet and if so for how long time, and (7) if the students were allowed to use their mobile phones in school and if so if they could connect to the school’s Wi-Fi network.

If the teacher in the questionnaire reported that he/she was near a microwave oven in the lunch room and there also was a peak signal at the frequency band 2.400–2.4835 GHz, we excluded the measurement data during that time. The signal from the oven (2.45 GHz) could be interpreted as a Wi-Fi signal by the exposimeter.

The information from the teachers, their work schedule, and questionnaire made it possible to relate different activities during the day with the measured values. The measurements took place during school time during the time period March–November 2016.

For the measurements of RF radiation, the exposimeter EME-Spy 200 from Satimo (MVG Industries, Brest, France) with a valid calibration was used. The exposimeter measures 20 predefined frequency bands, as presented in Table 2. These cover the frequencies of most public RF radiation emitting devices currently used in Sweden. The exposimeter covers frequencies of 87–5,850 MHz. For frequency modulation (FM), TV3, TETRA, TV4&5, Wi-Fi 2.4 GHz and Wi-Fi 5 GHz, the lower detection limit is 0.01 V/m (0.27 µW/m^2^); for all other bands, the lower detection limit is 0.005 V/m (0.066 µW/m^2^). For all bands, the upper detection limit is 6 V/m (95,544 µW/m^2^). The sampling time was every fourth second.

**Table 2:** Predefined measurement frequency bands of EME-Spy 200 exposimeter with frequency ranges.

Frequency band	Frequency minimum (MHz)	Frequency maximum (MHz)
FM	87	107
TV3	174	223
TETRA I	380	400
TETRA II	410	430
TETRA III	450	470
TV4&5	470	770
LTE 800, 4G (DL)	791	821
LTE 800, 4G (UL)	832	862
GSM 900 + UMTS 900, 3G (UL)	880	915
GSM 900 + UMTS 900, 3G (DL)	925	960
GSM 1800 (UL)	1,710	1,785
GSM 1800 (DL)	1,805	1,880
DECT	1,880	1,900
UMTS 2100, 3G (UL)	1,920	1,980
UMTS 2100, 3G (DL)	2,110	2,170
Wi-Fi, 2.4 GHz	2,400	2,483.5
LTE 2600, 4G (UL)	2,500	2,570
LTE 2600, 4G (DL)	2,620	2,690
WiMAX	3,300	3,900
Wi-Fi 5 GHz	5,150	5,850

*FM, frequency modulation; TV, television; LTE, long-term evolution; DL, downlink (transmission from base station to mobile phone); UL, uplink (transmission from mobile phone to base station); GSM, global system for mobile communications; UMTS, universal mobile telecommunications system; DECT, digital European cordless telecommunications; WiMAX, worldwide interoperability for microwave access*.

The exposimeter measures different telecommunications protocols: FM-radio broadcasting; TV broadcasting; TETRA emergency services (police, rescue, etc.); GSM second generation mobile communications; UMTS third generation mobile communications, 3G; long-term evolution (LTE) fourth generation mobile communications standard, 4G; digital European cordless telecommunications (DECT) cordless telephone systems standard; Wi-Fi 2.4 and 5 GHz wireless local area network (WLAN) protocol; worldwide interoperability for microwave access (WIMAX) wireless communication standard for high-speed voice, data, and Internet.

EME-Spy 200 utilizes 3-axis antenna to capture RF radiation from all possible directions. The unit reports the exposure after statistical processing, since each reported value is the sampling outcome, where many samples are collected and statistically processed including minimum, mean, median, and maximum values.

### Statistical Methods

Means, medians, minimum, and maximum values in microwatts per square meter were calculated for all measured frequency bands and for total exposure, and box plots were constructed to illustrate the distribution of total exposure for all teachers. Values below the lower detection limit were treated as null (0). Total exposure was calculated as the sum of all measured frequency bands at each measured data point. Stata/SE 12.1 for Windows (StataCorp., College Station, TX, USA) was used for all calculations.

## Results

In total, 18 teachers carried the exposimeter during one to four working days in school. Every teacher measured from 6 to 31 h resulting in 5,321–28,238 readings for each teacher. In total, 230,100 readings were assessed corresponding to 255 h of measurements of RF radiation. The total results for each teacher are displayed in Table 3 with mean, median, minimum, and maximum values for a total of all RF radiation exposures.

**Table 3:** Measurements of 18 teachers in seven schools in Sweden during March 14–November 10, 2016.

Teacher	*n*	Mean	Median	Min	Max
MH1	13,992	17.6	2.3	0.0	61,471.9
MH2	11,418	24.3	4.3	0.0	43,942.6
MH3	8,466	1.1	0.6	0.0	241.8
OH1	20,307	11.0	1.3	0.0	4,887.6
SH1	5,673	15.0	1.1	0.1	35,242.8
LG1	11,107	5.1	1.7	0.0	1,779.1
LG2	10,026	23.1	9.8	0.0	5,513.0
LG3	5,321	13.6	2.4	0.0	13,047.8
OG1	26,134	19.5	5.0	0.3	2,676.1
OG2	28,238	39.2	5.3	0.5	82,857.3
SG1	13,505	66.1	51.8	1.7	715.9
SG2	14,734	22.9	7.8	0.2	8,845.2
SG3	13,018	23.7	16.2	0.0	700.0
SG4	7,294	63.3	50.0	1.2	1,398.1
TG1	16,572	5.7	2.6	0.0	1,274.3
TG2	11,799	13.9	3.6	0.0	3,321.7
TG3	6,446	7.7	3.2	0.0	2,193.5
TG4	6,050	9.1	3.0	0.0	751.4
Total	230,100	22.5	4.6	0.0	82,857.3

*Results are given by teacher, analyses of all data, and total exposure (microwatts per square meter) treating values at detection limit as 0*.

**Figure 1:**
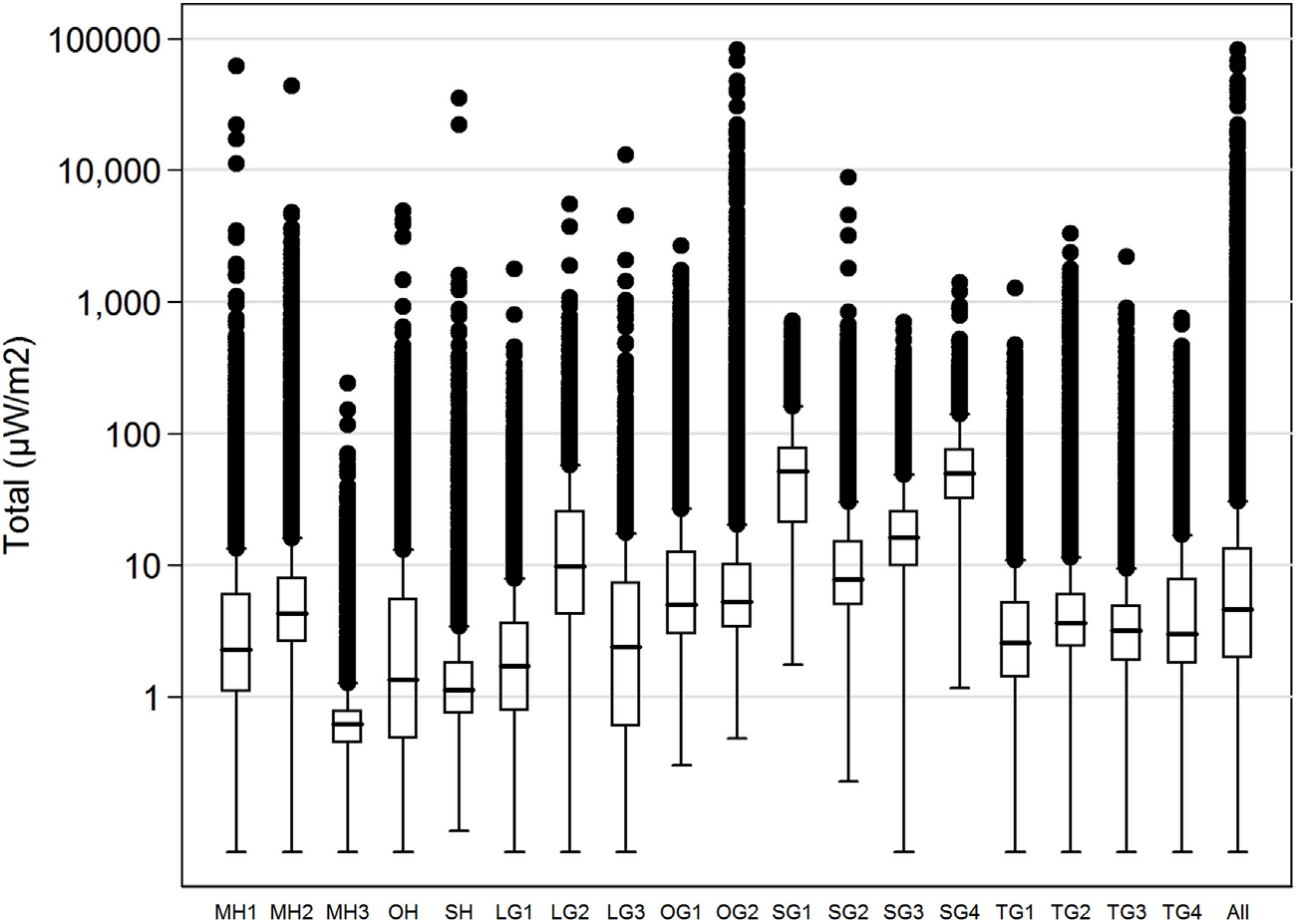
Boxplot, total exposure (microwatts per square meter), all 18 teachers, logarithmic scale. The median is indicated by the black line inside each box; the bottom and top of the boxes represent the first and third quartiles; the end of the whiskers is calculated as 1.5× interquartile range. The points represent the outliers.

The mean for each teacher ranged from 1.1 to 66.1 µW/m^2^ and medians from 0.6 to 51.8 µW/m^2^. The maximum values varied between 241.8 and 82,857.3 µW/m^2^. Figure 1 shows box plot for each of the 18 teachers with median values, boxes with the first and third quartiles and outliers. Table 4 shows all measurements divided into the 20 different frequency bands from 87 to 5,850 MHz. The highest peaks ranging from 43,938.7 to 82,856.6 µW/m^2^, came from mobile phones uplink from 4G 800 MHz, GSM 1,800 MHz, and 3G 2,100 MHz. The highest max downlink value, 3,285.9 µW/m^2^, came from 4G 800 MHz, while max for Wi-Fi 2.4 GHz was 4,482.8 µW/m^2^ and Wi-Fi 5 GHz 3,321.4 µW/m^2^. All separate means added up to a total of 22.5 µW/m^2^. Total median was 4.6 µW/m^2^.

**Table 4:** Measurements of 18 teachers in seven schools in Sweden during March 14–November 10, 2016, analyses of all data (microwatts per square meter) by frequency band treating values at detection limit as 0.

Frequency band	Mean	Median	Min	Max
FM	0.7	0.0	0.0	345.7
TV3	0.0	0.0	0.0	147.7
TETRA I	0.2	0.0	0.0	497.3
TETRA II	0.0	0.0	0.0	39.5
TETRA III	0.4	0.0	0.0	910.9
TV4&5	0.0	0.0	0.0	149.0
LTE 800, 4G (DL)	4.1	0.4	0.0	3,285.9
LTE 800, 4G (UL)	4.0	0.0	0.0	82,856.6
GSM + UMTS 900, 3G (UL)	0.2	0.0	0.0	2,874.5
GSM + UMTS 900, 3G (DL)	3.0	0.5	0.0	2,063.5
GSM 1800 (UL)	1.0	0.0	0.0	61,471.1
GSM 1800 (DL)	0.0	0.0	0.0	60.5
DECT	0.0	0.0	0.0	328.7
UMTS 2100, 3G (UL)	0.3	0.0	0.0	43,938.7
UMTS 2100, 3G (DL)	0.7	0.1	0.0	295.9
Wi-Fi 2.4 GHz	2.8	0.3	0.0	4,482.8
LTE 2600, 4G (UL)	0.3	0.0	0.0	3,768.9
LTE 2600, 4G (DL)	1.5	0.0	0.0	608.6
WiMAX	0.0	0.0	0.0	1.1
Wi-Fi 5 GHz	3.1	0.5	0.0	3,321.4
Total	22.5	4.6	0.0	82,857.3

*Totally 230,100 readings for each frequency band*.

**Figure 2:**
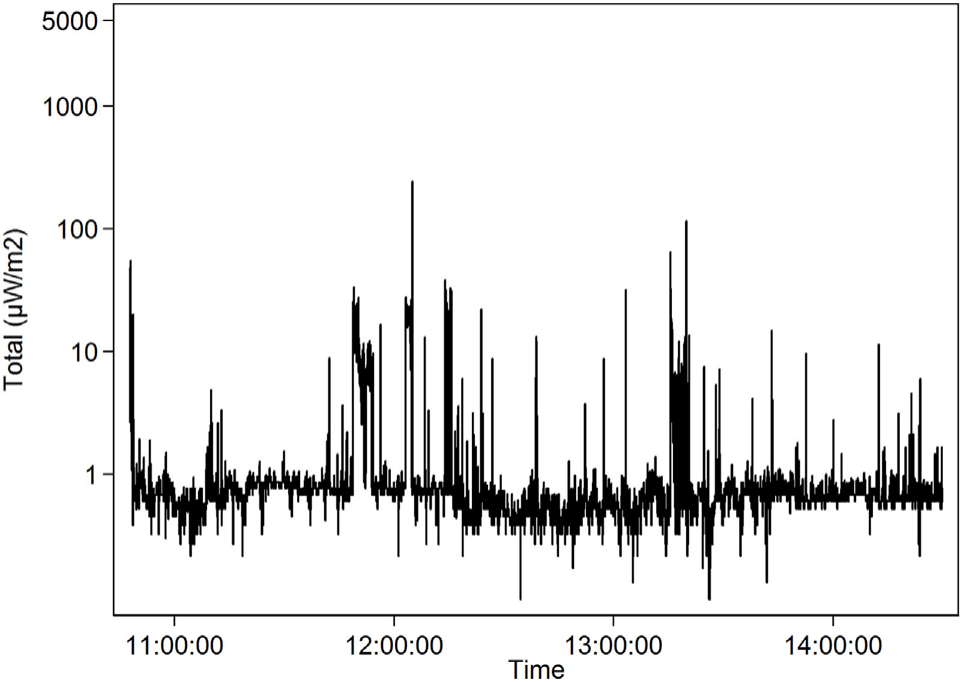
Teacher MH3 who had a wired connection to her laptop and the students used their laptops only in flight mode in the classroom. The teachers in the school were allowed to bear and use their own mobile phones, but not the students. Radiofrequency mean exposure 1.1 µW/m^2^ (logarithmic scale) over 1 day (November 9, 2016).

The teacher MH3 with the lowest mean, 1.1 µW/m^2^, taught in a classroom far from any Wi-Fi access point and had a wired connection to her own laptop for the education. The students used their laptops only in flight mode and mobile phones for students were banned during school time. The teachers in the school were allowed to carry and use their own mobile phones which can be seen by some higher peaks of GSM, 3G and 4G uplinks up to 151.1 µW/m^2^, Table 5 and Figure 2.

**Table 5:** Teacher MH3, November 9–10, 2016, analysis of all data (microwatts per square meter) by frequency band treating values at detection limit as 0.

Frequency band	Mean	Median	Min	Max
FM	0.0	0.0	0.0	52.7
TV3	0.0	0.0	0.0	0.3
TETRA I	0.0	0.0	0.0	0.0
TETRA II	0.0	0.0	0.0	0.0
TETRA III	0.0	0.0	0.0	0.9
TV4&5	0.0	0.0	0.0	0.0
LTE 800 (DL)	0.0	0.0	0.0	0.1
LTE 800 (UL)	0.0	0.0	0.0	31.5
GSM + UMTS 900 (UL)	0.0	0.0	0.0	13.0
GSM + UMTS 900 (DL)	0.5	0.5	0.0	14.9
GSM 1800 (UL)	0.1	0.0	0.0	151.5
GSM 1800 (DL)	0.0	0.0	0.0	1.7
DECT	0.0	0.0	0.0	1.0
UMTS 2100 (UL)	0.0	0.0	0.0	16.6
UMTS 2100 (DL)	0.1	0.0	0.0	3.2
Wi-Fi 2.4 GHz	0.3	0.0	0.0	111.5
LTE 2600 (UL)	0.1	0.0	0.0	129.6
LTE 2600 (DL)	0.0	0.0	0.0	0.9
WiMAX	0.0	0.0	0.0	0.0
Wi-Fi 5 GHz	0.0	0.0	0.0	2.1
Total	1.1	0.6	0.0	241.8

*Totally 8,466 readings for each frequency band. The teachers in the school were allowed to bear and use their own mobile phones, but not the students. The teacher had a wired connection to her laptop while the students only used their laptops in flight mode in the classroom*.

**Figure 3:**
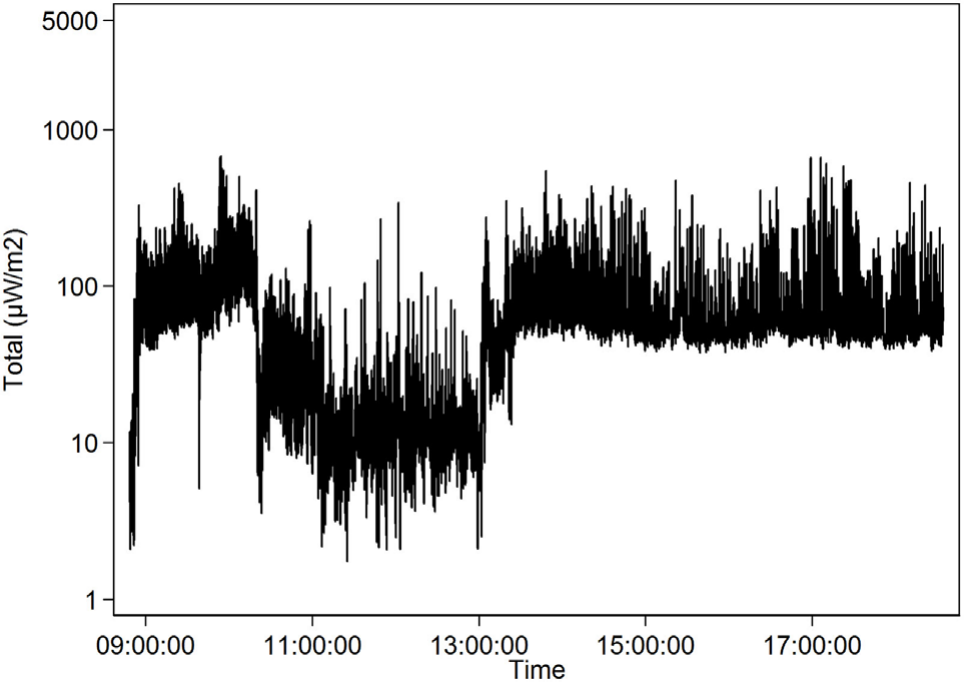
Teacher SG1. Total radiofrequency radiation (mean exposure 66.1 µW/m^2^, logarithmic scale) over 1 day (October 17, 2016). Results for a school with a weaker wireless fidelity network. Both students and teachers connected their mobile phones to base stations outside school.

The teacher SG1 with the highest mean 66.1 µW/m^2^ and median 51.8 µW/m^2^ taught in a school with a weaker Wi-Fi network which entailed that both students and teachers connected their mobile phones to base stations outside school. Here, the different mobile phone networks had a high impact on the total RF radiation exposure, see Table 6 and Figure 3. Exposure from Wi-Fi 2.4 and 5 GHz was low, partly because there was a wired connection to the teacher in each classroom.

**Table 6:** Teacher SG1, October 17–18, 2016, analysis of all data (microwatts per square meter) by frequency band treating values at detection limit as 0.

Frequency band	Mean	Median	Min	Max
FM	0.0	0.0	0.0	7.2
TV3	0.0	0.0	0.0	1.1
TETRA I	0.9	0.6	0.0	36.3
TETRA II	0.3	0.0	0.0	39.5
TETRA III	1.0	0.7	0.0	28.1
TV4&5	0.0	0.0	0.0	4.5
LTE 800 (DL)	27.5	16.6	0.0	676.5
LTE 800 (UL)	0.1	0.0	0.0	105.0
GSM + UMTS 900 (UL)	0.0	0.0	0.0	1.7
GSM + UMTS 900 (DL)	10.7	8.3	0.1	173.8
GSM 1800 (UL)	0.3	0.0	0.0	371.0
GSM 1800 (DL)	0.3	0.3	0.0	2.5
DECT	0.0	0.0	0.0	42.8
UMTS 2100 (UL)	0.0	0.0	0.0	8.3
UMTS 2100 (DL)	3.3	2.9	0.0	52.7
Wi-Fi 2.4 GHz	1.6	0.6	0.0	229.3
LTE 2600 (UL)	0.0	0.0	0.0	43.5
LTE 2600 (DL)	16.0	9.5	0.0	343.8
WiMAX	0.0	0.0	0.0	0.0
Wi-Fi 5 GHz	4.0	1.3	0.0	566.2
Total	66.1	51.8	1.7	715.9

*Totally 13,505 readings for each frequency band. Results for a school with a weaker wireless fidelity network. Both students and teachers connected their mobile phones to base stations outside school*.

**Figure 4:**
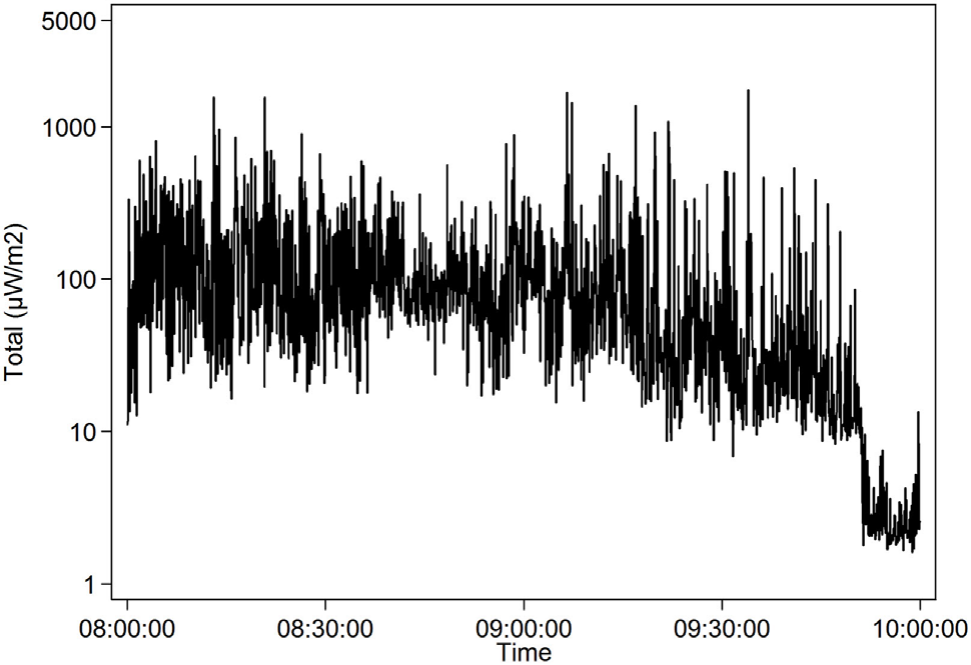
The highest mean level (107.3 µW/m^2^, logarithmic scale) during a whole lesson was recorded when the teacher OG1 stood right below an access point while using her wireless fidelity connected laptop (May 31, 2016).

**Figure 5:**
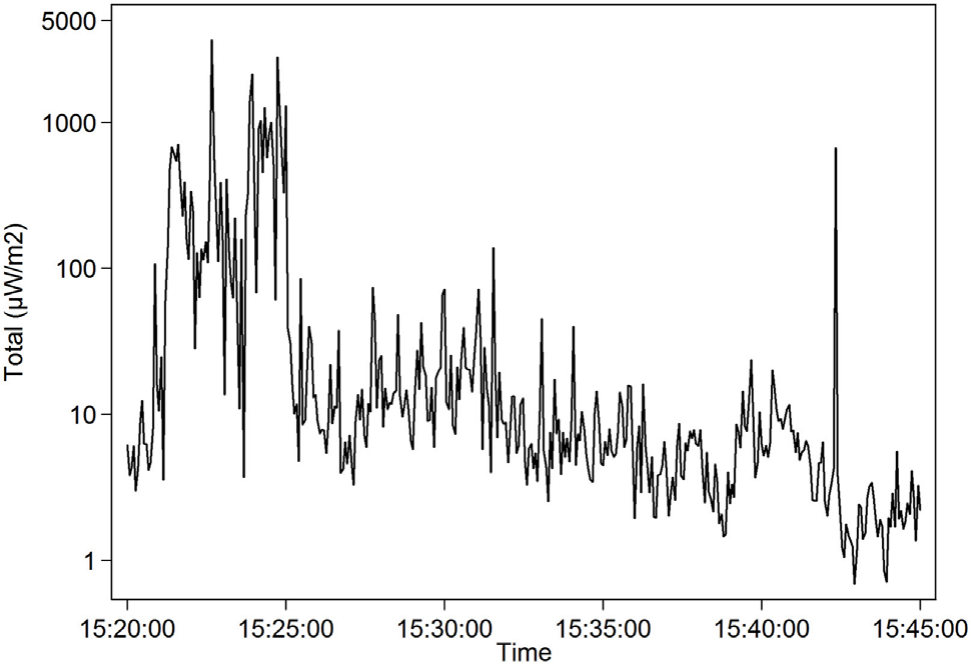
Teacher MH2 (November 8, 2016; 15:20–15:45, logarithmic scale). Radiofrequency radiation when students during a part of the lesson streamed and watched YouTube videos. For the time period 15:20–15:25, the mean exposure was 396.6 µW/m^2^.

The highest mean during a whole lesson, 107.3 µW/m^2^, is shown in Table 7 and Figure 4. The teacher OG1 stood right below an access point while using her Wi-Fi connected laptop during the lesson. None of the students used their laptops during this lesson. A short high mean came from a part of a lesson when another teacher, MH2, let the approximately 20 students in the class stream and watch YouTube videos. For a few minutes, the mean exposure went up to 396.6 µW/m^2^, mostly from Wi-Fi 2.4 GHz, Table 8 and Figure 5.

**Table 7:** Teacher OG1, May 31, 2016, 8:00–10:00, analysis of all data (microwatts per square meter) by frequency band treating values at detection limit as 0.

Frequency band	Mean	Median	Min	Max
FM	0.1	0.0	0.0	6.1
TV3	0.0	0.0	0.0	1.4
TETRA I	1.1	1.1	0.0	15.7
TETRA II	0.0	0.0	0.0	0.0
TETRA III	0.0	0.0	0.0	0.0
TV4&5	0.0	0.0	0.0	0.0
LTE 800 (DL)	1.5	1.3	0.1	14.1
LTE 800 (UL)	0.1	0.0	0.0	23.4
GSM + UMTS 900 (UL)	0.0	0.0	0.0	3.2
GSM + UMTS 900 (DL)	2.0	1.9	0.1	5.9
GSM 1800 (UL)	0.0	0.0	0.0	0.0
GSM 1800 (DL)	0.0	0.0	0.0	0.0
DECT	0.0	0.0	0.0	0.0
UMTS 2100 (UL)	0.0	0.0	0.0	0.4
UMTS 2100 (DL)	0.0	0.0	0.0	0.4
Wi-Fi 2.4 GHz	54.4	33.3	0.1	1,727.5
LTE 2600 (UL)	0.0	0.0	0.0	0.9
LTE 2600 (DL)	0.0	0.0	0.0	0.4
WiMAX	0.0	0.0	0.0	0.2
Wi-Fi 5 GHz	48.0	11.6	0.0	1,540.2
Total	107.3	62.7	1.6	1,742.3

*Totally 1,801 readings for each frequency band. The highest mean level during a whole lesson in this study was recorded when the teacher stood right below an access point while using her wireless fidelity connected laptop during the lesson*.

**Table 8:** Teacher MH2, November 8, 2016, 15:20–15:25, analysis of all data (microwatts per square meter) by frequency band treating values at detection limit as 0.

Frequency band	Mean	Median	Min	Max
FM	0.0	0.0	0.0	1.0
TV3	0.0	0.0	0.0	0.4
TETRA I	0.0	0.0	0.0	0.0
TETRA II	0.0	0.0	0.0	0.0
TETRA III	0.0	0.0	0.0	0.0
TV4&5	0.0	0.0	0.0	0.0
LTE 800 (DL)	0.0	0.0	0.0	0.0
LTE 800 (UL)	0.0	0.0	0.0	0.0
GSM + UMTS 900 (UL)	0.1	0.0	0.0	4.0
GSM + UMTS 900 (DL)	0.0	0.0	0.0	0.2
GSM 1800 (UL)	4.4	0.0	0.0	165.8
GSM 1800 (DL)	0.0	0.0	0.0	0.1
DECT	0.0	0.0	0.0	0.0
UMTS 2100 (UL)	0.0	0.0	0.0	1.1
UMTS 2100 (DL)	0.0	0.0	0.0	0.0
Wi-Fi 2.4 GHz	365.7	136.2	0.6	3,606.2
LTE 2600 (UL)	22.6	8.3	0.0	172.5
LTE 2600 (DL)	0.0	0.0	0.0	0.0
WiMAX	0.0	0.0	0.0	0.1
Wi-Fi 5 GHz	3.7	2.9	0.8	11.6
Total	396.6	153.8	3.0	3,650.3

*Totally 76 readings for each frequency band. The approximately 20 students in the class streamed and watched YouTube videos during a part of the lesson*.

## Discussion

### Short Summary of Our Results

Our measurements showed quite low-mean values from 1.1 to 66.1 µW/m^2^ with higher levels during lessons when laptops or mobile phones were actively used. The maximum values could be high when connecting to mobile phone base stations outside of the school building, up to 82,857.3 µW/m^2^ usually from uplink from GSM 1800, 3G or 4G. Maximum downlink from the same base stations was 3,285.9 µW/m^2^. Maximum peaks from Wi-Fi 2.4 GHz were 4,482.8 µW/m^2^ and from Wi-Fi 5 GHz 3,321.4 µW/m^2^. Total means for all teachers were for Wi-Fi 2.4 GHz 2.8 µW/m^2^ and for 5 GHz 3.1 µW/m^2^.

### RF Radiation Exposure in Sweden and in Schools in Other Countries

The levels of RF radiation have increased considerably during recent years both outdoor and indoor due to emergence of new telecommunication technologies and protocols. Measurements of outdoor exposure with a car-based measuring system done in 2013 in Sweden showed a median power density for RF fields between 30 MHz to 3 GHz to be 16 µW/m^2^ in rural areas, 270 µW/m^2^ in urban areas, and 2,400 µW/m^2^ in city areas (72). Areas with high exposure to RF radiation were measured with an EME-Spy 200 exposimeter in Stockholm Central Railway Station where the mean total RF radiation level varied between 2,817 and 4,891 µW/m^2^ (min 5.8, max 155,263.4 µW/m^2^) for each walking round (73). In the Old Town in Stockholm, the total RF radiation varied between a mean of 404 µW/m^2^ (min 20.4, max 4,088 µW/m^2^) on the streets around the Supreme Court, 756 µW/m^2^ (min 0.3, max 50,967 µW/m^2^) around the Royal Castle, and 24,277 µW/m^2^ (min 257, max 173,302 µW/m^2^) at Järntorget, a popular square with shops and outdoor restaurants (74).

A study was performed in a laboratory in the United Kingdom, which simulated a typical classroom with laptops but without any students. The laptops were set into the transmission mode and the exposure from 15 laptops and 12 Wi-Fi access points was measured. The measured maximum power density values were 22,000 µW/m^2^ at 0.5 m from the laptops and 87,000 µW/m^2^ at 0.5 m from access points decreasing to 4,000 and 18,000 µW/m^2^ at a 1 m distance, respectively (75).

A measurement study in schools in Belgium and Greece showed a total maximum value from all contributors of RF-EMFs of 1.6 V/m (6,790 µW/m^2^) and 1.66 V/m (7,310 µW/m^2^), respectively, and an average value of about 0.4 V/m (400 µW/m^2^) in both countries. In Belgium, it was mostly GSM 900 and the FM-radio signal that gave the highest exposure and in Greece GSM 1800 and 3G. None of the participating schools had education that involved the use of laptop by every student in the class. The exposure from Wi-Fi was low, in Belgium 6.6 µW/m^2^ and in Greece 21.4 µW/m^2^, Table 9 (76).

**Table 9:** Measurement studies in schools.

Reference	Country	Means for all RF radiation in µW/m^2^ (lowest and highest personal mean)	Means for Wi-Fi in µW/m^2^ (lowest and highest personal mean)	Means in µW/m^2^ for other RF sources (lowest and highest personal mean)
Vermeeren et al.^a^ (76)	Belgium Greece	403.6, mostly FM-radio and GSM 900 424.6, mostly GSM 1800 and 3G	6.6 21.4	FM 223.2 GSM 900 76.7 GSM 1800 193.4 UMTS 44.8
Verloock et al.^a^ (78)	Belgium	325.1	152.8	
Kurnaz et al.^a^ (81)	Turkey May 2016 June 2016 School with highest exposure	Mostly LTE 800, GSM 900,1800, UMTS 2100 229.2 171.3 29.944.7	1.5	
Karipidis et al. (83)	Australia		50–67
Body-borne exposimeters				
Valic et al.^a^ (77)	Slovenia Detection limit 6.6		9.5 (6.6–16.9)	GSM 13.0 (6.6–106.1) DECT 13.0 (6.6–152.8)
Gajšek et al.^a^ (80)	Slovenia	85.9	16.9	
Roser et al. (79)	Switzerland	59.7	2.2 (1.0–7.4)	
The present study	Sweden	22.5 (1.1–66.1)	2.4 GHz: 2.8 (0.2–8.9) 5 GHz: 3.1 (0–10.4)	Downlink forLTE 800 4.1 (0–27.5) GSM + UMTS 900 3.0 (0.1–28.1) UMTS 2100 0.7 (0–3.3) LTE 2600 1.5 (0–16.0)

*^a^In these papers, the radiofrequency (RF) radiation was measured in volts per meter. To make the measured values comparable electric field strength (*E*) in volts per meter have been converted to power density (*S*) in microwatts per square meter with the formula *S* = 0.002654 × *E*^2^ × 10^6^*.

In a study from 2010 to 2011 in Slovenia, 18 school children between the ages of 5 and 17 wore portable exposimeters day and night for an average of 69 h in total. High-maximum values of RF radiation were recorded for DECT, which exceeded the exposimeter’s upper measurable limit of 5 V/m (66,300 µW/m^2^), GSM with 3.46 V/m (31,800 µW/m^2^), and Wi-Fi 2.47 V/m (16,180 µW/m^2^). Most measurements of other frequencies for all the 18 children’s measurements showed low-average values, only barely over the detection limit on 0.05 V/m (6.6 µW/m^2^). The sample’s highest personal average value for Wi-Fi was 0.08 V/m (16.9 µW/m^2^) for GSM 0.20 V/m (106.1 µW/m^2^) and for DECT 0.24 V/m (152.8 µW/m^2^) (77).

A study in Belgium focused on schools with Wi-Fi and measured in five schools an average of 0.24 V/m (152.8 µW/m^2^) and peaks up to 3.21 V/m (27,347 µW/m^2^) for Wi-Fi signals (78).

In 2013–2014, 90 adolescents aged 13–17 years in Switzerland carried a portable exposimeter, ExpoM-RF, in a hip bag for 3 days. They also filled in a time-activity diary on a smartphone. The exposimeter measured every fourth second in 12 different frequency bands between 620 MHz and 2.4 GHz. Of the 90 students, 34 had WLANs in school and 86 of 90 had WLAN at home. All students had their own mobile phone and most, 86, had their own smartphone. Total average RF radiation exposure was 63.2 µW/m^2^. Main sources were from mobile phones (67.2%) and from mobile phone base stations (19.8%). WLAN at home and at school was only a small part of the personal exposure (3.5%), mean measurements in schools were 59.7 µW/m^2^, in trains 537.4 µW/m^2^, in busses 663.5 µW/m^2^, and in cars 832.2 µW/m^2^. In all these locations, mobile phones uplink of 900, 1,800, and 1,900 MHz were the main contributor. To have WLAN at home and at school showed lower total average exposure than with no WLAN at home and/or school (79).

In a study in Slovenia during 2014–2015, 49 pairs of child and parent carried an ExpoM-RF exposimeter for 3 days which also recorded geographic location using GPS. They also kept activity diaries on smartphones Average personal RF radiation exposure was 0.21 V/m (117 µW/m^2^) at home, 0.18 V/m (85.9 µW/m^2^) at school, and 0.31 V/m (255.0 µW/m^2^) at work. The main contributions from different sources were 0.11 V/m (32.1 µW/m^2^) from uplink, 0.18 V/m (85.9 µW/m^2^) from downlink, 0.15 V/m (59.7 µW/m^2^) from broadcasting, and 0.08 V/m (16.9 µW/m^2^) from WLAN (80).

In Turkey, measurements were made twice in May and June 2016 in 92 schools with PMM 8053 and Narda SRM-3006 exposimeters. The mean exposure was 0.2939 V/m (229.2 µW/m^2^) in May and 0.2541 V/m (171.3 µW/m^2^) in June. The main contributors were LTE 800 MHz, GSM 900 MHz, and GSM 1800 MHz. WLAN constituted a very small part with an average on 0.024 V/m (1.5 µW/m^2^) (81).

In a study in Melbourne, Australia in 2015, environmental RF radiation was measured in 20 different kindergartens and by 10 children aged 4–5 years who carried an exposimeter in a small bag attached around the chest. The exposimeter ExpoM-RF 64 could measure 16 frequency bands from 88 MHz to 5.8 GHz. There was a clear difference between kindergartens situated less than 300 m from base stations and those located more than 300 m away. The total median environmental exposure was 258 mV/m (176.6 µW/m^2^) for kindergartens situated less than 300 m from a base station compared with 75 mV/m (14.9 µW/m^2^) for those situated more than 300 m from a base station. The three highest contributors were GSM 900 MHz downlink and uplink and UMTS 2100 MHz downlink. The median personal exposure for all the 10 children was 81 mV/m (16.9 µW/m^2^) ranging from 31 mV/m (2.5 µW/m^2^) for the child with lowest exposure in a kindergarten more than 300 m away from a base station to 255 mV/m (172.5 µW/m^2^) for a child with the highest exposure in a kindergarten less than 300 m away from a base station (82).

Another study in Australia measured during 2016 in 23 schools with Wi-Fi networks with a Narda SRM-3006 Selective Radiation Meter with three separate axial probes from 9 kHz to 6 GHz. In only two schools, students were present and in one school a group of teachers were working with Wi-Fi devices. In the empty classrooms, one or more laptops were in active mode downloading large files or browsing the Internet. The average exposure, while walking through the classroom for 10 min, varied between 50 and 67 µW/m^2^ for Wi-Fi in idle or active conditions for the 20 schools and the maximum exposure varied between 840 and 1,100 µW/m^2^. The median duty cycle was for 2.45 GHz 6.3% and for 5 GHz 2.4%. The duty cycle is the proportion of time that Wi-Fi transmits RF signals. One conclusion of the study was that the personal exposure of each student to Wi-Fi will be largely dominated by the closest access point or client device rather than the total number of access points and devices around the school (83). Table 9 shows a comprehensive view of the measurements from the above studies compared with the present study.

The often short duty cycle in Wi-Fi networks can give low-average measured values but still consist of high peaks. Khalid et al. measured in schools in United Kingdom when students worked on web-based learning applications, surfed the Internet, downloaded files, and watched videos. The duty cycle for the children’s laptops varied from 0.02 to 0.91%, average 0.08%, and for the access points 1.0–11.7%, average 4.79%. The transmissions from Wi-Fi devices “consist of trains of pulses of RF energy ranging in duration from a few tenths of a milliseconds to about 10 ms, depending on the amount of data being carried by a burst.” Operating with maximal duty factors in a classroom with 30 laptops and an access point at a distance of only 0.5 m could give a maximal personal exposure of 16,600 µW/m^2^ (84).

Joseph et al. measured duty cycles of WLANs at 179 locations in urban areas, homes, and offices while the users surfed the Internet, downloaded YouTube videos, etc. The median duty cycle of all WLAN measurements was 1.4%. The actual duty cycle varied a lot depending on the load on the network and the network speed. The maximum measured duty cycle was 93.6% for file transfer over a slow network. Surfing and audio streaming require less Mbits and have lower duty cycles, usually below 3.2% (85).

The different modulations result in signals with different spectral characteristics within the channel for the device. If these modulations would be shown to be important variables in accessing risk, the need for RF radiation protection would probably change (86).

The importance to minimize the users’ exposure to the EMFs while retaining the network connectivity was discussed in a study with different wireless devices. Connection to the network *via* Enhanced Data rates for GSM Evolution or General Packet Radio Services technologies showed the strongest fields measured with a 14-point measurement model on the body. WLAN connection or 3G within excellent network reception showed significantly less exposure (87). Another study showed higher mean and maximum values for 3G/4G, especially with long distances to base stations (88).

### Limitations Due to Method of Measurements

Our results were based on limited numbers and should therefore be interpreted with caution. For definite conclusions larger studies are needed including more schools and teachers.

The present study used an EME-Spy 200 exposimeter for measurements, see also our earlier studies (73, 74). Because samples were taken every fourth second, technologies with large differences between mean and peak might not have been exactly evaluated, an inherent limitation of the exposimeter, which is intended to determine the dosage. For example, exposures from the Wi-Fi access points may have been undervalued with the used exposimeter. Generally, peak signal level measurement data are interesting when discussing the non-thermal health effects of RF radiation. Also the duty cycle, that was not possible to measure in our study, is of importance. A short duty cycle, even if the RF radiation is very high during emission, will usually give a low-mean value. A long duty cycle will more correctly correspond to the peaks in the transmission and give a higher mean value.

The shielding effect from the body of a person carrying an exposimeter can be considerable when comparing a body-borne exposimeter with an exposimeter mounted on a car roof (89), or compared with an exposimeter placed in the middle of a room (90). This has to be considered when analyzing any exposimetric measurements. To use an exposimeter with three-axis antenna, like the EME-Spy 200 in the present study, may minimize body shielding according to Bhatt et al. (91).

The teachers carrying the exposimeter are used as proxies for the students’ exposure in schools. They often move around in the classroom and may in this way have been exposed to both higher and lower values of RF radiation. Depending on where access points are located and how long time the connection to the Internet is used, the exposure can differ between students sitting nearby each other using laptops. The teachers’ exposure is also valuable to know since they are exposed for as long as the students each day. A case study describes both students and teachers, who have shown symptoms and become sensitive to RF radiation from Wi-Fi sending devices in school (92).

### Risk Management

The measurements show that letting the students connect their own mobile phones to the school’s Wi-Fi network decreases the exposure levels compared with connecting mobile phones to GSM, 3G and 4G base stations. To not allow students to bring mobile phones to school or collect these in turned off mode every morning decreases the exposure even more.

It is essential to consider the duty cycle—the period during which the Wi-Fi devices are sending. A high-effective Wi-Fi network minimizes the duty cycles, the time the children are exposed and the average exposure value while the students use the Internet, but the background RF radiation may be higher with high peaks and this may influence well-being, especially for electromagnetic hypersensitive persons. The highest peaks in our study (see Table 4) came from the mobile phone’s uplink to the base stations outside school. If the teacher or student keeps their mobile phone in the pocket close to the body, the exposure can be considerable every time the mobile phone connects for updating data. Activated Wi-Fi in mobile phones usually connects very frequently to the Internet (often every 5–10 s).

The largest impact in reducing students’ exposure to wireless radiation can be observed when wired network connections are used instead of wireless connections. While wireless connections are the cause for the students’ exposure to RF radiation, wired connections are not accompanied by such radiation. Even when wired connections are used in computers, wireless connections may still be active and in use by students’ and teachers’ mobile phones. A strategy to reduce the exposure to RF radiation would require (1) removing mobile phones from the learning environment or (2) to switch these into flight mode, which deactivates all wireless transmissions or (3) to switch off the wireless connections on the phone (Wi-Fi, mobile data), and leaving on only the mobile telephony voice communication connection (GSM or UMTS or LTE).

To download large files and stream videos, as for the students of teacher MH2, see Figure 5, will give high exposure to RF radiation. In contrast, surfing on the Internet or working mostly on already downloaded programs gives lower exposure. The RF radiation from Wi-Fi could be minimized by using wired connections for both the teachers and students.

Higher exposure to wireless radiation was also encountered in classrooms where Wi-Fi access points were positioned in the classroom as in contrast to schools where the access points were positioned further away from the students. An exposure reduction solution would be to place the access points outside from classrooms. The RF radiation may have to be stronger, though, if the signals from the Wi-Fi access point outside a classroom have more difficulty to reach the clients’ devices through a thick wall of concrete. Also the radiation from the access point should be directed only toward the client’s device, relieving other people in the vicinity from the unnecessary radiation.

The teacher OG1 in Figure 4 and Table 7, standing right under an access point while using a laptop during the lesson led to higher Wi-Fi exposure compared with the schools which had wired connection to the Internet for the teacher in the classroom (Tables 5 and 6).

Since the wireless radiation is always active, even if the devices are not in use, this also contributes to the exposure budget of the students and teachers. An option to reduce the exposure would be to activate the wireless access points only for the period when the network connection is really needed for study purposes.

Several of the schools participating in our measurement study were situated in small towns or outside the city center. The environmental exposure from base stations located at the surrounding buildings (downlink) was low compared with our measurements in Stockholm city (73, 74). Thus, with low-outdoor exposure there will be less RF radiation indoors. Reducing children’s exposure to RF radiation would require removing mobile phone base station antennas from adjacent buildings. In case of finding locations for new schools, these should be located away from the mobile phone base stations.

Students’ use of mobile devices for social networking and other non-study activities that are irrelevant for learning goals during school time may contribute to a significant portion of the wireless communications. An exposure reduction solution would call for rules disallowing the usage of wireless connections for any other use than school-related tasks.

A few smaller schools in Sweden use only wired connection to the Internet. More and more schools have decided to ban mobile phones during the school day to avoid distraction during lessons and to increase social interactions in breaks. Thereby also the RF radiation is reduced in the school. It is important to get all the advantages from ICT and in the same time prevent any potential long-term health effects. The teacher MH3 in Figure 2 had the lowest mean, median, and maximum values in our study. She had lessons in a classroom far from Wi-Fi access points, students’ mobile phones were not allowed, the teacher’s laptop had a wired connection, and the students’ laptops were in flight mode.

There is a complex picture about well-being and the use of laptops, tablets, and smartphones in schools and at home. It is unclear to what extent the screen time, the more sedentary activities, multitasking, lack of sleep, and changing of everyday life due to the technical development, that influence the physical and mental well-being. However, the RF radiation from wireless devices may also have a smaller or more pronounced impact on the well-being of children and adolescents. There seems to be a big difference in sensibility to RF radiation between individuals both among humans and animals in studies (92, 93).

Since children are obliged by law to attend school, the safe environment with respect to physical hazards is of utmost importance. Our study showed low-average exposure compared with our measurements in city streets and squares in Stockholm where the average exposure often is around 1,000 µW/m^2^. All measured values in the schools were far below ICNIRP’s reference values (26), but most total mean measured levels were above the precautionary target level of 3–6 µW/m^2^ as proposed by the Bioinitiative Report (28). It is unclear whether it is the average level that have the most impact on health or if the peaks and the length of the duty cycle can affect cell systems more in the human body. Since cancer tumors usually take several decades to develop and chronic illnesses, like cardiac and neurological diseases, come in older ages only the future will tell if and to what degree the RF radiation may have had an impact on these illnesses.

More worrying for today is the increase in behavioral problems in children like ADHD, the increasing sleep problems among children, and mental illnesses with anxiety, depressed mood, and suicide thoughts (11, 17–23, 25, 53–56). Children are probably more sensitive to RF radiation because of their growing bodies and more immature cells, but also because they will be exposed throughout their life-time (41, 44, 45).

Given the abundance of microwaves in the modern environment, it is of importance that children grow up in an electromagnetic safe environment. The scientific research this far gives no guarantees for safety from the RF radiation. Children should be exposed to as low-RF radiation as possible both in school and at home. Wired connection for both the Internet and telephone communications should be preferred to minimize children’s exposure to the wireless radiation, Table 10.

**Table 10:** The following actions are examples of methods to reduce children’s exposure to RF radiation in schools.

Wired connection to both teachers and students and no wireless networks or devices in school is the optimal choice. If this is not possible: Wired connection to each classroom to the teacher’s laptop, for the students to download large files and videos. To reduce exposure from Wi-Fi networks in school: turn off Wi-Fi access points when not used for learning purposes, position Wi-Fi access points outside of classrooms, use directional Wi-Fi access points, which radiate into the direction of the client’s device. Keep laptops and tablets in flight mode when Internet is not needed for learning purposes. Wired connection to a landline telephone in each classroom could minimize the need for mobile phones for contact. Mobile phones, including smart phones, could be left at home or collected in turned off mode. If allowed, they should be carried only in flight mode during school hours.

In Conclusion

1. The ICNIRP guidelines are based on short-term heating (thermal) effects, and are therefore not relevant to decide on the appropriateness of long-term exposure.
2. The environmental exposure to RF radiation in some schools is higher than reported levels for non-thermal biological effects. In order to reduce children’s exposure to RF radiation, schools should prefer wired network connections, allow laptop, tablets, and mobile phone usage only in flight mode and deactivate Wi-Fi access points when internet is not needed for learning purposes.

## Ethics Statement

This study was carried out in accordance with the recommendations of The Ethical Committee, Uppsala University, with written informed consent from all subjects: all subjects gave written informed consent in accordance with the Declaration of Helsinki. The protocol was approved by The Ethical Committee, Uppsala University, Sweden (DNR 2015/485).

## Author Contributions

LKH and LH made contributions to the conception and design of the study. LKH, LH, and TK handled the measurements and information to schools and teachers. TK contributed with technical advice for the exposimeters and the measurements. MC made the statistical analyses, the tables, and the figures. All the authors agreed on the risk management principles. LKH drafted the manuscript. All authors revised the manuscript and gave approval to the final version to be published.

## Conflict of Interest Statement

The authors declare that the research was conducted in the absence of any commercial or financial relationships that could be construed as a potential conflict of interest. The reviewer HM and handling editor declared their shared affiliation.
